# Characteristics of Phytoplankton Biomass, Primary Production and Community Structure in the Modaomen Channel, Pearl River Estuary, with Special Reference to the Influence of Saltwater Intrusion during Neap and Spring Tides

**DOI:** 10.1371/journal.pone.0167630

**Published:** 2016-12-01

**Authors:** Weihua Zhou, Jie Gao, Jianzu Liao, Ronggui Shi, Tao Li, Yajuan Guo, Aimin Long

**Affiliations:** 1 Key Laboratory of Tropical Marine Bio-resources and Ecology, and Guangdong Provincial Key Laboratory of Applied Marine Biology, South China Sea Institute of Oceanology, Chinese Academy of Sciences, Guangzhou, China; 2 Tropical Marine Biological Research Station in Hainan, South China Sea Institute of Oceanology, Chinese Academy of Sciences, Sanya, China; 3 South China Business College, Guangdong University of Foreign Studies, Guangzhou, China; 4 South China Sea Environment Monitoring Center, State Oceanic Administration, Guangzhou, China; 5 University of Chinese Academy of Sciences, Beijing, China; Fisheries and Oceans Canada, CANADA

## Abstract

In recent decades, increasing frequency and intensity of saltwater intrusion in the Modaomen Channel has threatened the freshwater supply in the surrounding cities of the Pearl River Estuary, and ulteriorly changed the environmental conditions of the estuarine waters. Phytoplankton biomass, primary production (PP) and species composition, as well as hydrological and chemical parameters were examined along a downstream transect in the Modaomen Channel during neap tide (NT) and spring tide (ST), when a strong saltwater intrusion event occurred in late September, 2011. A total of 46 species phytoplankton were identified, including Bacillariophyta (25 species), Dinoflagellate (14 species), Chlorophyta (4 species), Cyanophyta (2 species) and Euglenozoa (1 species). The dominant species were shifted from freshwater diatoms (e.g., *Melosira granulata* and *Melosira granulata* var. *angustissima*) in the upper reaches to saline water diatoms (e.g., *Skeletonema costatum* and *Coscinodiscus* sp.) in the river mouth. Generally, phytoplankton density, biomass (chl-*a*) and PP decreased from the upper to lower reaches along the channel, and were significantly higher in NT than those of ST. There was a shift from large-sized phytoplankton (>20 μm) in the upper reaches to relative small-sized cells (5–20 μm) in the lower reaches. Compared to NT, low discharge and flow velocity, coupled with strong easterly winds during ST specially aggravated saltwater intrusion further to the upstream (~50 km from the estuary). The intruded saltwater diluted nutrients, N/P ratios, chl-*a*, and phytoplankton abundances, and thereby led to a decline in PP during ST.

## Introduction

Tidal dynamics play a critical role in affecting estuarine biological production, water quality, material transport and dispersion [1, 2]. Saltwater intrusion, a universial phenomenon in estuaries, is the movement of saline water into freshwater aquifers that partly results from tidal action, and has a profound effect on the estuarine ecosystem and environment [2]. Saltwater intrusion plays an important role in the distribution of chemical and biological variables in estuarine waters [3, 4]. Saltwater intrusion can result in reduction of species diversity [5, 6], and hence seriously impact the structure of the biological communities [7]. In recent decades, saltwater intrusion has become more frequent and severe, and impaired the regional water supply along the Modaomen Channel, Pearl River Estuary (PRE) [8]. It can be mainly attributed to the increase in freshwater demand, the decline in discharge in the upper reaches, the increase in tidal amplitude at the river mouth, the rising sea level, and channel deepening due to sand mining [9, 10].

Phytoplankton are the base of the aquatic food chain, and are important producers in most estuarine and coastal waters [11]. They can rapidly respond to environmental changes. For example, phytoplankton production may rely on nutrient concentrations and a suite of physical factors, such as vertical mixing, changes in flushing and residence time, and altered optical properties in estuarine and coastal systems [12]. The seasonal characteristics of phytoplankton community, biomass and primary production in the PRE were described by Huang et al. [13, 14], Yin et al. [15–17], Qiu et al. [18], Li et al. [6] and Shen et al. [19], as well as their environmental regulations. They indicated that the physical environment and nutrient levels were directly related to river discharge resulting in the succession of phytoplankton taxa. However, to date, limited studies have been conducted on phytoplankton community characteristics, biomass and primary production, as well as their regulation mechanisms during neap tide (NT) and spring tide (ST) when saltwater intrusion occurred in the PRE. The saltwater intrusion in the Modaomen Channel usually occurs during the dry season from October through to April [20]. According to a report on Zhongshan Daily, saltwater intrusion event began in early August 2011, which began two months earlier than the previous years (e.g. October in 2010) [21]. Strong saline water intruded into the Quanlu Water Plant located in the upper reaches of the Modaomen Channel in mid-September [22]. This provided us an ideal opportunity to compare the different spatial distrubution patterns of phytoplankton community structure, biomass, size structure and primary production based on two cruises during the NT and ST in mid- and late September coincident with a strong saltwater intrusion event. The main influencing factors of different intrusion distance between NT and ST were compared. The effects of the saltwater intrusion intensity on the characteristics of nutrients and phytoplankton were also discussed.

## Materials and Methods

### Study area and water sampling

The Pearl River is the second largest river in China and ranks the 13th in terms of annual freshwater discharge in the world, which is 3,360 × 10^8^ m^3^ [16]. It has three major branches in the upper drainage basin, i.e. the West River (Xijiang), the North River (Beijiang), and the East River (Dongjiang) [23]. The West River is the largest and contributes 77% of water discharge and 86% of suspended sediment [24]. The Modaomen Channel, with the largest discharge among the eight major outlets in the PRE, is the main part in the lower reaches of the West River [8]. The Modaomen Channel is an important freshwater source for cities in the western Pearl River Delta, such as Zhongshan, Zhuhai and Macau. However, the freshwater supply is greatly restricted by saltwater intrusion, especially during the dry season when saltwater intrudes upstream frequently [25].

**Fig 1:**
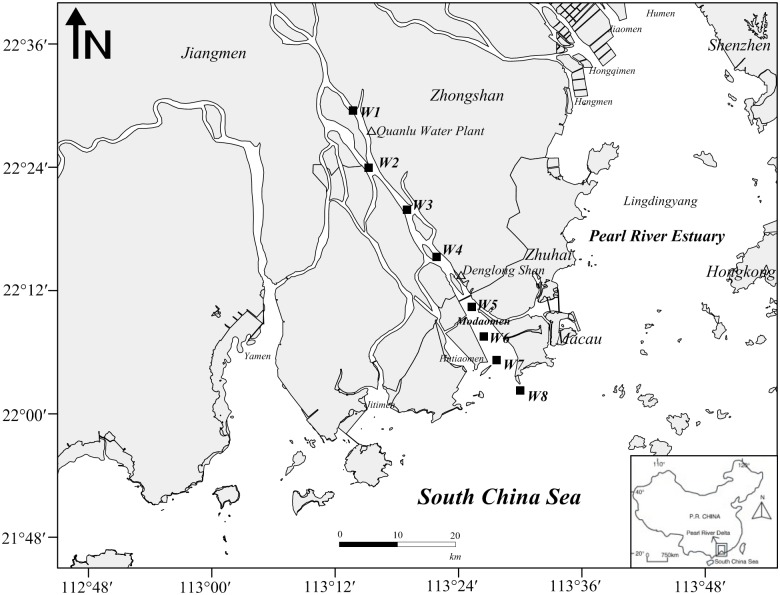
The location of sampling stations along the transect from W1 to W8 in Modaomen Channel, Pearl River Estuary.

Samples of nutrients, phytoplankton biomass and community composition, and primary production were collected along an eight stations downstream transect (4 in upper reaches, Stns W1 to W4, and 4 in lower reaches, Stns W5 to W8) in the subtropical Modaomen Channel, PRE (with irregular semi-diurnal tide) during NT (September 18–20) and ST (September 25–27) in 2011 under the permission of Guangdong Provincial Oceanic and Fishery Administration, China (Fig 1 and Table 1). Water samples were collected from surface (1 m below the surface) and bottom (1 m above the bottom) by a Niskin bottle attached to a nylon cable.

**Table 1:** The depth, longitude and latitude of sampling stations.

Stations	Depth (m)	Longitude (°E)	Latitude (°N)
W1	8	113.229	22.492
W2	9	113.254	22.399
W3	13	113.316	22.331
W4	9	113.364	22.255
W5	8	113.421	22.173
W6	11	113.440	22.126
W7	9	113.462	22.087
W8	6	113.500	22.038

### Measurements

#### Meteorological and hydrological parameters

Water temperature and salinity were measured with a SEACAT CTD system (SBE21, Sea-Bird Electronics Inc., Washington, USA). The daily freshwater discharge were obtained from Gaoyao Gauging Station in the West River (http://xxfb.hydroinfo.gov.cn/). Flow velocity were determined by an Aanderaa Current Meter (RCM9, Aanderaa Instruments Inc., Bergen, Norway) at the anchor station W5 with 1 h interval measurement during the course of the whole NT and ST. The daily total rainfall, the prevailing average daily wind speed and direction in September 2011 were obtained from the Waglan Island Automatic Weather Station of the Hong Kong Observatory (HKO), which is located on the east side of PRE (http://www.weather.gov.hk/).

#### Nutrients

Nutrient Samples (NH_4_, NO_3_, NO_2_, PO_4_ and SiO_4_) were filtered through acid-cleaned 0.45 μm cellulose acetate filters and were kept frozen until analyzed in laboratory. Nutrients were determined according to the Specification for Oceanographic Survey of Chemical Parameters in Sea Water [26]. Dissolved inorganic nitrogen (DIN, is defined as NH_4_ + NO_3_ + NO_2_) and PO_4_ concentrations were used to calculate atomic ratios of N/P.

#### Phytoplankton biomass (chl-*a*)

Water samples (150 mL) for total phytoplankton biomass (chl-*a*) were passed through 0.7 μm Whatman GF/F filters under low-vacuum pressure (<100 mm Hg). Samples of 300 mL for size fraction chl-*a* were sequentially filtered through 20 μm, 5 μm Poretics polycarbonate membranes and Whatman GF/F filters to collected >20 μm 5–20 μm, and <5 μm size fraction chl-*a*, respectively. Filters containing chl-*a* were extracted with 10 mL 90% acetone and sonicated for 10 min in an ice-cold water bath, and then, extracted at 4°C in the dark for 24 h. The fluorescence of the extract was measured using a Turner Designs 10-AU Fluorometer, which was precalibrated using Sigma pure chl-*a* [27].

#### Primary production

Water samples for primary production (PP, ^14^C uptake rates) incubations were prescreened through a 200 μm mesh net, and then transferred to 50 mL Kimax^®^ tubes, inoculated with 2 μCi labeled sodium bicarbonate (NaH^14^CO_3_). The tubes were wrapped with different neutral density screens to simulate the irradiance levels corresponding to different water sampling depths. Samples were incubated for 4–6 h between 08:00 and 16:00 h after being placed horizontally in a water bath flushing with surface water on board the ship to maintain *in situ* temperature. After the incubation, samples were filtered through Whatman GF/F filters, and the filters were kept frozen (-20°C) until they were analyzed within one month following the JGOFS protocols by a Beckman LS6500 Liquid Scintillation Counter [28].

#### Species composition and abundance

Sub-samples for phytoplankton taxonomic composition and abundance (each 1000 mL) were immediately preserved in approximately 1.5% acidic Lugol's solution and stored in polycarbonate bottles before being treated in laboratory. For each sample a 50 mL sub-sample was concentrated using Utermöhl Settling Chamber for at least 24 h. Supernatant was aspirated so that the sample was concentrated to 1 mL [19]. The concentrated sample at the bottom area of the whole chamber was then examined under an Olympus BX51 microscope (Olympus, Japan) to identify the species composition and to enumerate the abundance of phytoplankton by following the method of Utermöhl [29].

### Data and statistical analysis

According to Haralambidou et al. [4], water column stratification in each sampling station along the Modaomen Channel was assessed by the stratification index (*N*), which is defined as:

$$N=\delta S/S_{0}$$ (1)

Where, *δS* is the salinity difference between surface and bottom, *S*_*0*_ is the vertically averaged salinity. When N <0.1, the water column is completely mixed, when 0.1< N <1.0 then partial mixing occurs, while if N >1.0 stratification with the presence of saltwater intrusion is evident.

Phytoplankton biological diversity (*H'*), evenness (*J*) and the dominance index (*Y*) were calculated according to Shannon and Wiener [30] and Pielou [31]:

$$\;H^{\prime }=-\overset{S}{\underset{i=1}{\sum }}P_{i}{\text{log}}_{2}\;P_{i}\;(P_{i}=n_{i}/N);$$ (2)

$$J=\frac{H^{\prime }}{{\text{log}}_{2}^{}S}$$ (3)

$$Y=(n_{i}/N)\times f_{i}$$ (4)

Where, *n*_*i*_ is the individual amount of different phytoplankton species, *N* is total individual amount, *S* is the total species, and *f*_*i*_ is the appearance frequency at all of the stations investigated. Combined with contribution (%) of total cell density, a subjective constant of the dominance index *Y* (≥0.02) was adopted in this study as the criterion for a dominant species [32].

Figures were constructed by Surfer 8.0 (Golden Software Inc.) and SigmaPlot 12.5 (SPSS Inc.). Data were analyzed by SPSS 13.0. Pearson correlation analysis was applied and probabilities (*P*) of <0.05 were considered to be significant.

## Results

### Meteorological and hydrological conditions

**Fig 2:**
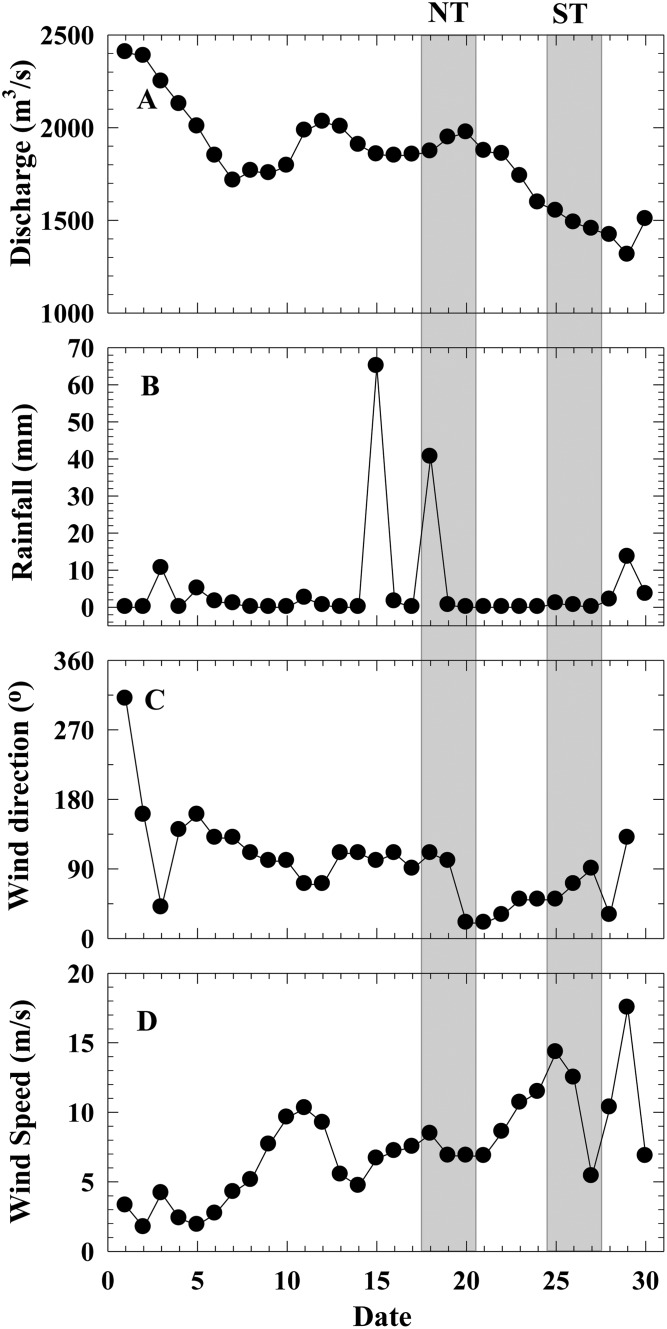
Daily discharge (A), total rainfall (B), average wind direction (C) and wind speed (D) during September 2011. The lighter gray areas indicate sampling period during neap tide (NT, the same below) on Sep 18–20, and spring tide (ST, the same below) on Sep 25–27.

Freshwater discharge decreased gradually in September. The average discharge rate was 1,929 m^3^/s and 1,497 m^3^/s during NT and ST sampling period, respectively (Fig 2A). The total rainfall was only 149 mm in September, and 105 mm precipitated during September 15–18 just before and among NT sampling period. Only 3.5 mm rainfall was recorded during ST (Fig 2B). The prevailing daily wind direction was easterly in September (Fig 2C). The wind speed increased from early to late September, averaged 3.7 m/s, 7.0 m/s and 9.7 m/s in early, mid- and late September, respectively (Fig 2D).

**Fig 3:**
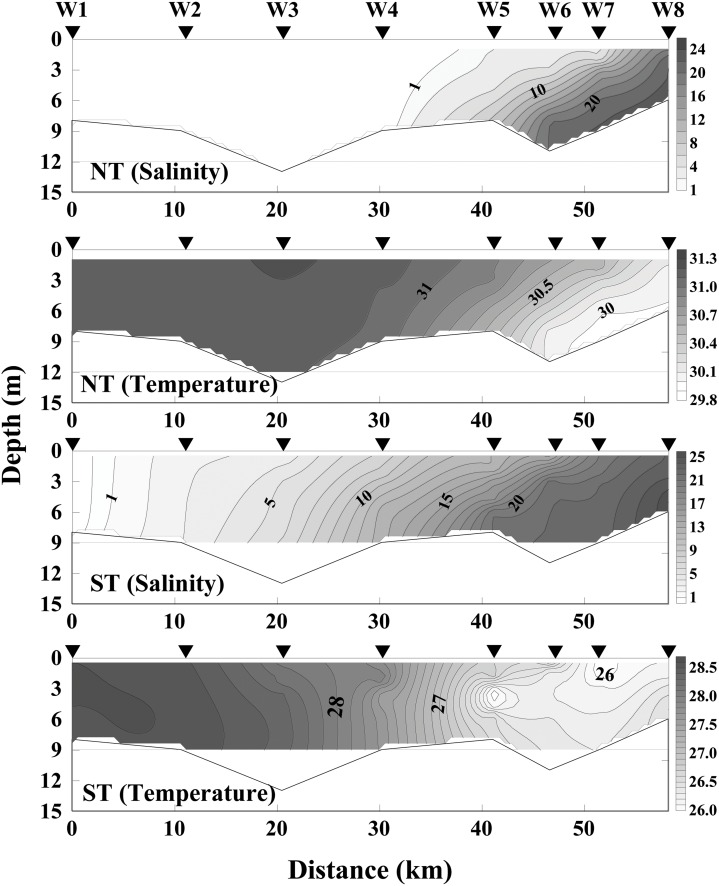
Depth contours of salinity (psu) and temperature (°C) at NT and ST along the transect W1-W8 in the Modaomen Channel. Arrows indicate the positions of the stations where vertical profiles were taken.

During NT, water column salinity ranged from 0.1 to 22.1 psu, and temperature ranged from 30.0 to 31.2°C along the channel. Temperature and salinity were almost homogeneous in the whole water column in the upper reaches (Stns W1 to W4) without saltwater intrusion effect (Fig 3). In contrast, the lower reaches were obviously intruded (~25 km from the estuary) by saltwater with relatively lower temperature (~30°C). Bottom salinity were >20 psu from Stns W6 to W8. Strong stratification occurred in the lower reaches of the channel (Fig 3).

During ST, the water column salinity varied from 0.1 to 25.5 psu, and temperature varied from 25.9 to 28.6°C. Compared to NT conditions, the formation of thermocline and halocline was limited along the channel during ST. A gradient in water temperatures was observed from Stn W1 (~28.6°C) to Stn W8 (~26°C) along the channel (Fig 3). Saltwater intruded near the Quanlu Water Plant (~50 km from the estuary) between Stns W1 and W2 (Figs 1 and 3).

### Nutrients

**Fig 4:**
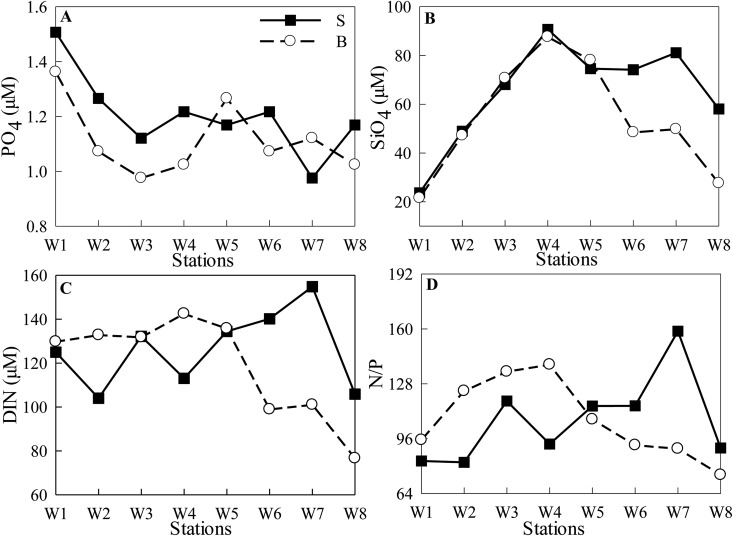
Nutrients concentrations (A: PO_4_, B: SiO_4_, C: DIN) and N/P ratios (D) of the transect at the surface (S, the same below) and bottom (B, the same below) during NT.

During NT, average PO_4_ concentrations (± SD) were 1.20 ± 0.15 μM (ranged from 0.98–1.51 μM) at the surface and 1.12 ± 0.13 μM (ranged from 0.98–1.36 μM) at the bottom, respectively. PO_4_ decreased from Stns W1 to W3, and varied slightly from Stns W4 to W8 (Fig 4A). SiO_4_ increased from Stn W1 (~20 μM) to Stn W4 (~90 μM), and then decreased along the channel (Fig 4B). DIN concentrations were >100 μM, except at the bottom water of Stn W8 (~75 μM). Atomic DIN:PO_4_ (N/P) ratios were generally >80:1 at the surface and >90:1 at the bottom (Fig 4D).

**Fig 5:**
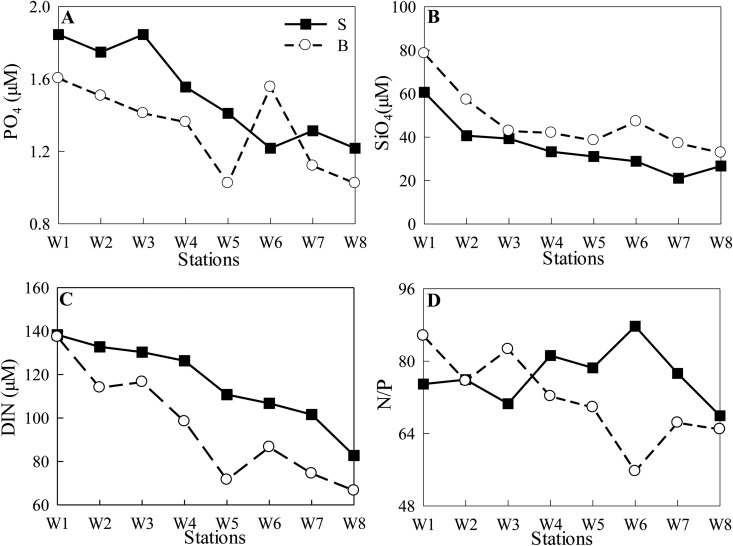
Nutrients concentrations (A: PO_4_, B: SiO_4_, C: DIN) and N/P ratios (D) of the transect during ST.

In contrast, the spatial distribution of all species of nutrients significantly decreased along the channel during ST (Fig 5). Surface PO_4_ and DIN were much higher than those at the bottom. The fact that surface SiO_4_ were lower showed that in addition to runoff, another possible source of SiO_4_ was mainly from sediment resuspension. Atomic N/P ratios were generally >64:1 at all stations (Fig 5D).

### Phytoplankton biomass (chl-*a*)

**Fig 6:**
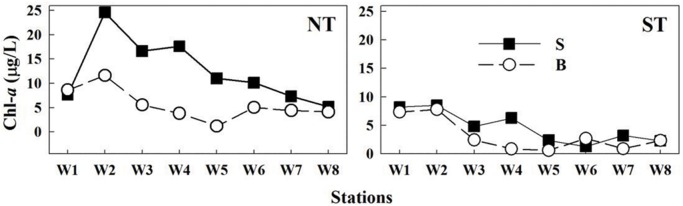
Chl-*a* concentrations of the transect during NT and ST. Note the change in the scale on the y-axis.

In general, chl-*a* decreased from the upper reaches to the estuary, and surface chl-*a* was generally higher than bottom, except at Stn W6 during ST, which was consistent with the nutrient distribution pattern at this station (Figs 5 and 6). Surface chl-*a* was >5 μg/L in the upper reaches. At the shallow Stn W8, chl-*a* was uniform in the water column due to increased mixing (Fig 6). Average chl-*a* at the surface and bottom during NT were 12.5 ± 6.6 μg/L and 5.6 ± 3.2 μg/L, respectively. While during ST, chl-*a* were 4.6 ± 3.1 μg/L at the surface and 4.1 ± 2.5 μg/L at the bottom.

**Fig 7:**
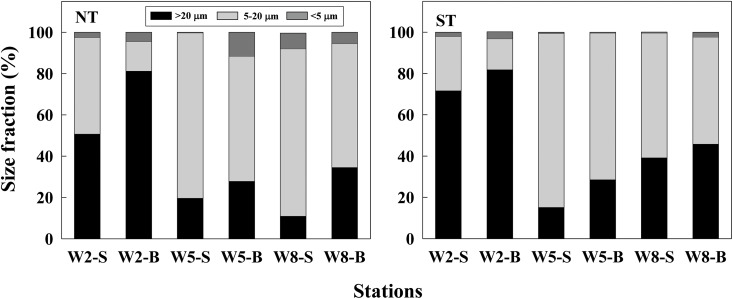
Contribution (%) of different size fractions to the total phytoplankton biomass (chl-*a*) along the transect at surface (S) and bottom (B) during NT and ST.

The results of size fractionated chl-*a* showed that >20 μm size cells contributed the main part of phytoplankton in the upper reaches, which accounted for up to 50% and 70% at the surface during NT and ST, respectively, and had a much higher proportion at the bottom. While the 5–20 μm fraction dominated at lower reaches. Only a very small part of phytoplankton biomass (<10%) was attributed to the <5 μm size cells along the transect both during NT and ST (Fig 7). The results indicated that there was a shift from larger size phytoplankton in freshwater zones compared to smaller cells in estuarine waters.

### Primary production

**Fig 8:**
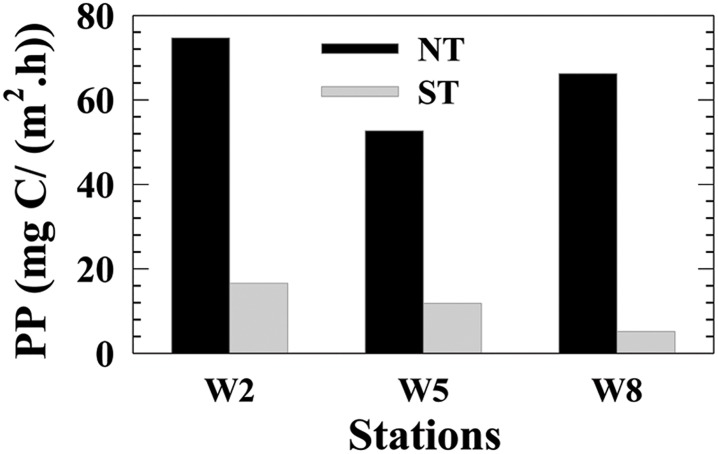
Primary productivity (PP, mg C/(m^2.^h)) of the transect during NT and ST.

Water column PP was significantly higher during NT than during ST (Fig 8). Similar to phytoplankton biomass, the spatial distribtion of PP showed that higher reates in the upper reaches of the channel. Water column PP was >50 mg C/(m^2^·h) during NT, and <20 mg C/(m^2^·h) during ST. Average PP was 64.5 ± 11.1 mg C/(m^2^·h) during NT, compared to a ~6-fold reduction in PP (11.2 ± 5.7 mg C/(m^2^·h)) during ST.

### Species composition and abundance

A total of 46 species of phytoplankton were identified, including Bacillariophyta (diatom, 25 species), Dinoflagellate (14 species), Chlorophyta (green algae, 4 species), Cyanophyta (2 species) and Euglenozoa (1 species). In the whole region, the dominant species were mainly freshwater diatoms, such as *Melosira granulata* (*Y* = 0.69), *Melosira granulata* var. *angustissima* (*Y* = 0.07). Cell abundances of *Melosira gramulata* were >80% at Stn W2 in the upper reaches during NT and ST conditions, and decreased seawards along the channel (Table 2). Saline water diatoms dominated at the river mouth, e.g., *Skeletonema costatum* (Greville) Cleve dominated at the estuarine Stn W8 during NT and *Coscinodiscus subtilis* var. *minorus* dominated at the lower reaches during ST (Table 2, S1 Table).

**Table 2:** Phytoplankton cell density, biological diversity (*H'*), evenness (*J*) and dominant species.

Tide	Station	Layer	Cell Density (×10^4^ cells/L)	*H'*	*J*	Dominant species (% of total cell density)
**NT**	W2	S	52.63	0.75	0.20	*Melosira granulata* (83.8%), *Melosira granulata* var. *angustissima* (14.6%)
**NT**	W2	B	21.89	0.79	0.21	*Melosira granulata* (85.7%), *Melosira granulata* var. *angustissima* (11.1%)
**NT**	W5	S	9.53	1.07	0.27	*Melosira granulata* (83.1%), *Melosira granulata* var. *angustissima* (8.2%)
**NT**	W5	B	2.77	1.19	0.34	*Melosira granulata* (75.8%), *Melosira granulata* var. *angustissima* (17.7%)
**NT**	W8	S	0.32	2.79	0.81	*Skeletonema costatum* (34.4%), *Coscinodiscus subtilis* var. *minorus* (9.4%)
**NT**	W8	B	0.20	3.25	0.94	*Skeletonema costatum* (20%), *Gymnodinium catenatum* (15%), *Prorocentrum* sp. (15%)
**ST**	W2	S	12.77	0.66	0.19	*Melosira granulata* (88.3%), *Melosira granulata* var. *angustissima* (9.4%)
**ST**	W2	B	14.26	0.60	0.18	*Melosira granulata* (88.1%), *Melosira granulata* var. *angustissima* (10.9%)
**ST**	W5	S	0.24	2.86	0.86	*Gymnodinium catenatum* (37.5%), *Coscinodiscus subtilis* var. *minorus* (13.2%)
**ST**	W5	B	0.38	2.23	0.70	*Melosira granulata* (55.3%), *Coscinodiscus subtilis* var. *minorus* (13.2%)
**ST**	W8	S	0.13	2.87	0.96	*Coscinodiscus subtilis* var. *minorus* (23.1%), *Coscinodiscus* sp. (15.4%), *Thalassiosira sp*. (15.4%), *Prorocentrum triestinum* (15.4%)
**ST**	W8	B	0.36	3.23	0.90	*Melosira granulata* (22.2%), *Coscinodiscus subtilis* var. *minorus* (16.7%), *Bacillaria paradoxa* (13.9%)

NT, Neap Tide; ST, Spring Tide; S, surface; B, bottom.

According to cell density, diatoms dominated the phytoplankton assemblage (97%), with smaller contributions from dinoflagelate 1.3%, Chlorophyta 0.8%, Cynophyta 0.7%, and others <0.1%. Cell density significantly decreased from upper to lower reaches along the channel (Table 2, S1 Table). Average abundance of phytoplankton varied from 20.8 × 10^4^ cells/L at surface to 8.3 × 10^4^ cells/L at bottom during NT, while during ST the average abundance ranged from 4.4 × 10^4^ cells/L at surface to 5.0 × 10^4^ cells/L at bottom.

Phytoplankton biological diversity (*H'*) increased significantly along the Modaomen Channel, from 0.75 to 3.25 (average 1.64 ± 1.09) and from 0.60 to 2.87 (average 2.08 ± 1.16) during NT and ST, respectively. In contrast, evenness (*J*) decreased along the channel, from 0.20 to 0.94 (average 0.46 ± 0.33) during NT, and from 0.18 to 0.96 (average 0.63 ± 0.36) during ST (Table 2).

## Disscusion

### Stratification and saltwater intrusion dynamics: NT *versus* ST

**Fig 9:**
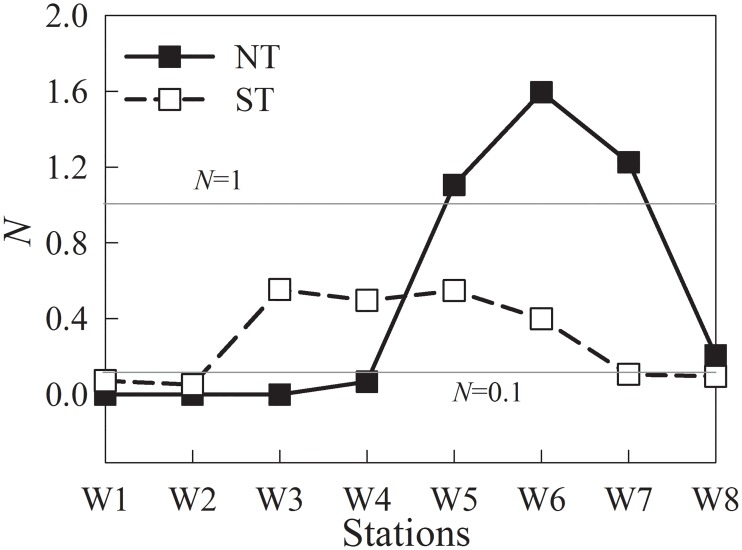
Stratification indexes (*N*) of the transect during NT and ST.

**Fig 10:**
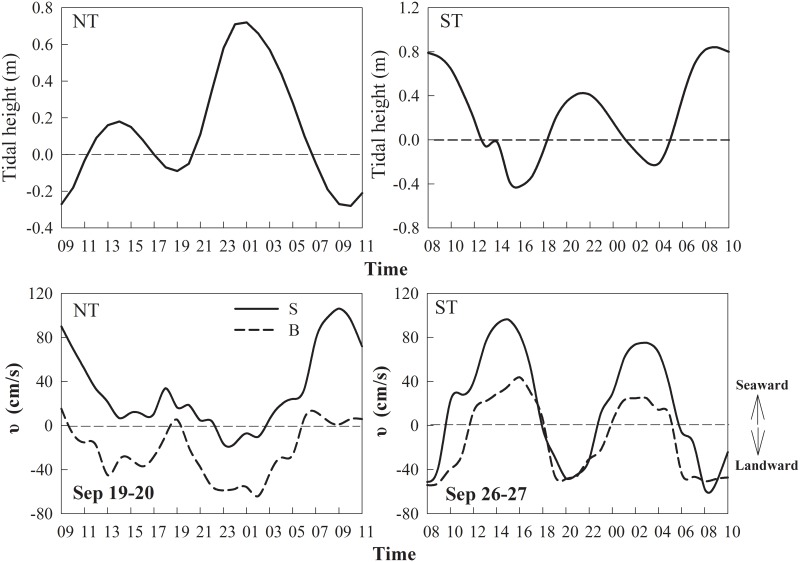
Tidal height (m) and flow velocity (υ, cm/s) variation during NT and ST at the anchor Stn W5.

Stratification is a common phenomenon in estuarine regions with a less dense freshwater surface and deeper saltwater layers that can result in significant intrusions due to physical forcing and tidal influences [2]. It has been identified as an important mechanism in the spatial and temporal regulation of phytoplankton community and primary production in estuarine systems [33]. To some degree, stratification can enhance the driving baroclinic pressure gradient and the gravity current, and hence aggravate saltwater intrusion to upper reaches [20]. Commonly, the maximum salinity occurs during NT compared to ST events [25]. This was not the case in the present study, and the specific drivers were evaluated in the present study. The stratification indexes calculated in Fig 9 indicated that NT saltwater intrusion induced strong stratification in the lower reaches of the Modaomen Channel, except Stn W8 (*N* = 0.2), where the bathymetry (with shallow water depth 6 m) was probably one of the main factors. In contrast, the whole water column was partially mixed from Stns W3 to W6 (Fig 9) during ST. The different stratification intensity of NT and ST also coincided with changes in flow velocity at the anchor station W5 (Fig 10). During the neap tide cycle, surface flow was in the direction of the ocean. Exceptions occurred at high water periods with freshwater discharge that resulted in saltwater intrusion events upstream. In contrast, during the spring tide cycle, both surface and bottom flow directed seaward during low water period and opposite during high water period.

Estuarine dynamics are controlled by riverine, marine, meteorological and geomorphologic factors [34]. The distribution and species succession of phytoplankton were also greatly influenced by the dynamics of the salt wedge [35]. Normally, the saltwater intrusion in Modaomen Channel appears during the dry season, and is further enhanced from November to next February. Moreover, saltwater intrusion could arrive in advance due to low discharge caused by extreme weather event [22]. Compared with the other waterways in the Pearl River Delta, the physical and dynamic mechanisms of seawater intrusion in Modaomen Channel exhibit an opposite pattern [20]. It was shown that saltwater intrusion began in 2–3 days before the NT. Then, saltwater intruded upstream quickly with an increasing tide range, and reached its maximum in moderate tide about 2–3 days before the ST when the mixing between freshwater and seawater tended to uniform vertically. Finally, the seawater was brought out until the ST with a strong tidal dynamic role [8, 25, 36, 37]. There are two NT and ST in a month due to moon movement, meaning that the saltwater intrudes at least twice a month. Bao et al. observed that two intensive saltwater intrusions occurred in the Modaomen Channel in the second half of month, but its short residence time did not cause severe impacts due to a large tidal range and forcing. The findings of the study indicated more significant impacts during NT than ST, given higher residual seawater in the upstream areas [36, 38]. They attributed this abnormal saltwater intrusion to the combined effect of small net downstream water transport and special current variation during NT [36]. Wang et al. suggested increased saltwater in the Hongwan Waterway, a narrow and shallow channel connecting the Modaomen Channel with the sea, was spilled over into the Modaomen Channel during NT or the coming moderate tide [25]. But this was not the case in our present study.

Modaomen is a micro-tidal channel [39]. The saltwater intrusion intensity (distance) in the Modaomen Channel are mainly regulated by freshwater discharge in the upper reaches, tidal amplitude at the river mouth [9, 40], river flow [8], wind direction and speed [41, 42]. In micro-tidal estuaries, river discharge plays a dominant role in controlling the development and advance or retreat of a salt-wedge. Increased levels of river flow tend to move the salt-wedge towards the ocean, while low levels of discharge can result in saltwater intrusion long distances upstream [4]. For example, the Swan, Pearl and Yura River estuaries, the salt wedge is forced downstream or eroded at times of high freshwater output [1, 23, 43]. For the Modaomen estuary, Gong and Shen [8] concluded that there is a consensus that saltwater intrusion is inversely correlated to river discharge. A high river discharge results in a reduced saltwater intrusion and vise versa. When the river flow decreased, a net landward transport of seawater is generated. The prevalence of NE/E winds helps to push the saltwater upstream along the Modaomen Channel [25, 42, 44]. According to Scully et al. [45], more intense winds directed upstream of the estuary tend to reduce vertical stratification. Compared to NT conditions in our study, relatively low discharge, strong easterly winds and low river flow velocity (Table 3) likely contributed to enhanced saltwater intrusion during ST.

**Table 3:** Averaged freshwater discharge, prevailing wind direction, wind speed and surface flow velocity (seaward).

Tide	Discharge (m^3^/s)	Wind direction	Wind speed (m/s)	Flow velocity (cm/s)
NT	1929	E	7.3	32.1
ST	1497	E	10.7	16.1

NT, neap tide; ST, spring tide; E, eastward.

### Impact of saltwater intrusion on the distribution of nutrients and phytoplankton

**Fig 11:**
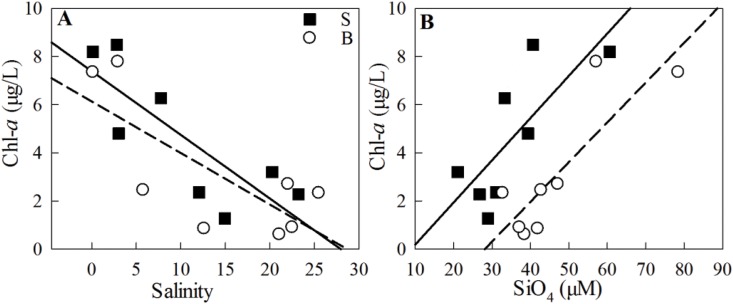
Relationship of salinity and chl-*a* (A), SiO_4_ and chl-*a* (B) during ST. In figure (A), the liner regression correlation coefficients (R^2^) were 0.66 (*P* < 0.01) and 0.56 (*P* < 0.05) at surface (solid line) and bottom (dash line), respectively. In figure (B), the correlation coefficients (R^2^) were 0.58 (*P* < 0.01) and 0.72 (*P* < 0.01) at surface and bottom, respectively.

Biological processes are strongly coupled with physical processes [23]. Physical dynamics, such as nutrient-depleted oceanic saltwater intrusion, play a critical role in the regulation of estuarine biological species composition, biomass and production, material transport and consequently, water quality [1]. As reported by Haralambidou et al. [4], saltwater propagation strongly affects the distribution of water quality parameters along the Strymon River estuary, Northern Greece. The results of the present study indicated reduction in bottom nutrient concentrations and N/P ratios during NT events along the channel in the stratified lower reaches (Fig 4). In contrast, during ST, the entire transect was diluted by oceanic saltwater intrusions. As a result, the bio-chemical parameters were significantly correlated with salinity, except surface N/P ratios (Table 4, Fig 11A).

**Table 4:** Correlations (R^2^, with *P* values) between salinity and bio-chemical parameters during ST in the whole transect (W1-W8).

Layer	chl-*a*	DIN	PO_4_	SiO_4_	N/P ratio
S	0.66 (*P*<0.01)	0.96 (*P*<0.0001)	0.88 (*P*<0.001)	0.72 (*P*<0.01)	0.04 (*P*>0.05)
B	0.56 (*P*<0.05)	0.93 (*P*<0.0001)	0.53 (*P*<0.05)	0.62 (*P*<0.05)	0.75 (*P*<0.01)

S, surface; B, bottom; DIN, dissolved inorganic nitrogen.

The salinity changes, through the mixing of freshwater and seawater, affect biota in the lower reaches of rivers and in estuaries [46]. Under sufficient tidal exchange processes, the lower channel and estuary were dominated by marine sources, even during high freshwater discharge periods. As a result, nutrient concentrations and phytoplankton biomass in the lower channel were effectively diluted by contributions from oceanic waters. This resulted in a shift from freshwater to saline water diatoms along the estuary axis [14, 47, 48]. Huang et al. [14] and Zhang et al. [47] showed that typical riverine phytoplankton, *Melosira granulata* and *Melosira granulata* var. *angustissima*, with a limited salinity tolerance, were the dominant freshwater diatoms. While *Skeletonema costatum* was the dominant estuarine species in the PRE. They also found lots of freshwater diatoms even appeared in the lower reaches during the wet season when no saltwater intrusion occurred. *Skeletonema costatum* is a pollution-tolerant and indicator species of eutrophication, which can adapt to high N:P conditions [49]. In our study, the biomass of *Skeletonema costatum* decresaed in the estuary during ST, indicating the low-nutrient seawater intrusion may play an importance role. Interestly, we found the freshwater *Melosira granulate* made up a small proportion at the stn W8 during ST, possibly carried form the upper reaches by the strong tidal mixing during ST. Biomass of *Coscinodiscus subtilis* var. *minorus* was higher at stns W8 and W5 during ST compared to NT, likely related to a higher tolerance to lower nutrient levels compared to freshwater forms. In general, our results suggested saltwater intrusion in Modamen Channel will result in obligate freshwater phytoplankton transfer to the euryhaline or saline water species to the middle even the upper channel regions.

Moreover, salt-wedge intrusion appears to control the estuarine supply of nutrients, turbidity induced light penetration changes and temperature, thus regulating the spatial characteristics of phytoplankton primary production [1, 4, 50, 51]. Vertical mixing not only diluted the phytoplankton biomass by mixing the algal cells throughout the water column but also resulted in light limitation by transporting the phytoplankton cells out of the euphotic zone [52]. Compared with NT, the relatively low chl-*a* and primary production during ST were attributed to higher vertical mixing to a certain extent.

### Nutrient regulation on phytoplankton biomass

According to atomic N/P ratios (mostly >64:1), P could be potentially limited for phytoplankton growth. Due to the continuing input of high river discharge, nutrient concentrations were always maintained at high levels during both ST and NT, with DIN >60 μM, PO_4_ >1.0 μM, and SiO_4_ >20 μM (Figs 3 and 4). Based on the calculated kinetics of nutrient uptake by Justić et al. [53], the concentration threshold of DIN, PO_4_ and SiO_4_ for all phytoplankton growth had been estimated to be 1.0 μM, 0.1 μM and 2.0 μM, respectively. Hence, nutrient concentrations were not actually limiting the growth of phytoplankton in the Modaomen Channel, except SiO_4_ at Stn W1 during NT and at estuarine stations (Stns W7 and W8) during ST.

PO_4_ concentration declined due to *Melosira granulata* blooms (>80% of the phytoplankton abundance, mostly chl-*a* >15 μg/L during NT, and >6 μg/L during ST) in the upper reaches during NT and ST (Figs 4A and 5A). SiO_4_ significantly correlated with chl-*a* both at surface and bottom waters during ST (Fig 11B), indicating the importance of diatom taxa.

## Conclusions

In the present study, a total of 46 species phytoplankton were identified during NT and ST in Modaomen Channel, PRE, including Bacillariophyta (25 species), Pyrrophyta (14 species), Chlorophyta (4 species), Cyanophyta (2 species) and Euglenophyta (1 species). The dominant species were mainly freshwater diatoms, such as *Melosira granulata* and *Melosira granulata* var. *angustissima*. Compared with previous studies, some saline water species (e.g., *Skeletonema costatum*, *Coscinodiscus* sp.) dominated in the river mouth and even appeared at the middle channel due to strong saltwater intrusion during ST[14, 19, 47]. Cell size structure showed that there was a shift from larger size freshwater diatoms (>20 μm) to smaller brackish water diatoms (5–20 μm, such as *Coscinodiscus subtilis* var. *minorus*) from the upper to lower reaches. With the increasing intensity of saltwater intrusion, the phytoplankton growth was likely inhibited by high saline oceanic waters with lower nutrients in the bottom. According to our results, saltwater intruded further upstream during ST (~50 km) than during NT (~25 km), mainly due to heavy rainfall yielding high river discharge and flow velocity, as well as relatively weak easterly wind during NT. Phosphorus potentially limited phytoplankton biomass in the Modaomen Channel. During ST, the increased intensity of saltwater intrusion diluted nutrient concentrations, N/P ratios, chl-*a* biomass, phytoplankton abundances, and thereby lead to a decline in PP.

## Supporting Information

S1 Table Phytoplankton species composition and abundance of the transect during NT and ST. (XLS)
